# *In Vitro* Screening for Cytotoxic Effect of Pore Forming Colicin N and Its Domains on Human Cancer Cells

**DOI:** 10.21315/tlsr2022.33.1.10

**Published:** 2022-03-31

**Authors:** Methawee Duangkaew, Wanatchaporn Arunmanee

**Affiliations:** Department of Biochemistry and Microbiology, Faculty of Pharmaceutical Sciences, Chulalongkorn University, Bangkok, 10330, Thailand

**Keywords:** Pore Forming Toxin, Colicin, Cytotoxicity Study, Protein Therapeutics, Bacteriocins

## Abstract

Protein-based drugs have increasingly become an important segment of cancer treatment. In comparison with chemotherapy, they offer high efficacy and fewer side effects due to specifically targeting only cancer cells. Monoclonal antibodies are currently the main protein-based drugs in the market but their complexity and limitations in tumour penetration led to the development of alternative protein therapeutics such as pore-forming toxins. Colicin N (ColN), a pore-forming protein produced by *E. coli*, was previously found to exhibit cytotoxicity and selectivity in human lung cancer cells with promising potential for further development. Here we aimed to screen for the cytotoxicity of ColN in breast (MCF-7 and MDA-MB-231), lung (A549) and colon cancer cells (HT-29 and HCT-116) by MTT (3-(4,5-Dimethylthiazol-2-yl)-2,5-Diphenyltetrazolium Bromide) assay with various concentrations for 72 h and to investigate the cytotoxic effect of ColN domains on cancer cells. It showed that ColN mildly mediated the decrease in cell viability except for MCF-7. The highest effect was seen in A549 and HCT-116 cells which showed 31.9% and 31.5% decrease in cell viability, respectively. The mild inhibition or promotion of cancer cell proliferation by ColN tend to be based on the cell types. Furthermore, to search for the functional domain of ColN used for cytotoxicity, full-length ColN and truncated ColN with deletion of translocating, receptor binding and pore-forming domains were also tested on HCT-116 colon cancer cells. The findings indicated that HCT-116 cells were not significantly sensitive to ColN but full length ColN caused slight decrease in cancer cell viability. The data in this study will benefit the further development of ColN for alternative protein-based cancer therapy.

**Highlight**

- The full-length and truncated colicin N with the correct folding were successfully expressed in *E. coli* and purified.
- Colicin N exhibited the mild toxicity against human breast (MDA-MB-231), lung (A549) and colon cancer cells (HT-29 and HCT-116). The toxicity level of ColN depends on the type of cancer cell lines.
- All three domains of colicin N are required for the cytotoxic effect of colicin N on human colon cancer cells (HCT-116).

## INTRODUCTION

Chemotherapy, among several other cancer therapeutic approaches, is currently a conventionally standard treatment for cancer patients. However, chemotherapeutic agents tend to cause many side effects due to their lack of specificity to cancer cells. Furthermore, cancer cells can develop resistance to the current chemotherapeutic agents by increasing their expression of drug detoxifying enzymes and drug transporters or they can repair DNA defects to stop apoptosis (Raguz & Yagüe 2008). The treatment of cancers with high efficacy and fewer side effects is needed to reduce patient mortality rates and improve their quality of life.

Protein-based therapeutics are a promising alternative to efficiently treat cancers while minimising harmful side effects. Monoclonal antibodies (mAb) are among the key protein-based therapeutic drugs in the pharmaceutical industry prescribed for cancer treatment (Lagassé *et al*. 2017). In 2019, of the top ten drugs by sales globally, five were mAb related cancer drugs (Urquhart, 2020). However, because of their highly complex structure and post-translational modifications (Samaranayake *et al*. 2009), the production of mAb requires a costly mammalian cell expression system (Dingermann 2008) and complex purification process (Liu *et al*. 2010). Furthermore, the tumour penetration of mAb is limited due to their large size (Minchinton & Tannock 2006). To overcome these limitations, several classes of toxins have been produced and tested as an alternative for cancer therapeutic agents. Pore forming toxins from sea anemones, bacteria or humans have shown a cytotoxic effect against leukemic cells (Wan *et al*. 2006), breast cancer cells (Avila *et al*. 1989), lung cancer cells (Avila *et al*. 2007, Mutter *et al*. 2018) and colon cancer cells (Tejuca *et al*. 2009). These toxins can be easily produced by recombinant technology. Moreover, in the era of biotechnology, these toxins can be genetically engineered to fuse with either fragments of antibodies or growth factors with the aim to enhance cancer target therapy (Kintzing *et al*. 2016). The production of these hybrid toxins could be achieved in microbial expression systems which are more cost-effective and highly scalable when compared to the production of mAb in mammalian cell expression system. This would make this alternative protein-based therapy more accessible to developing countries.

Colicin is a bacteriocin produced and secreted by *Escherichia coli (E. coli*) to kill other susceptible Gram-negative bacteria in times of stress. In addition to its antibacterial activity, it has been reported that colicins, especially pore-forming colicins, possess anticancer activities against several cancer cell lines such as breast, bone and colon cancer (Kaur & Kaur 2015, Chumchalova & Smarda 2003, Smarda *et al*. 2001). Colicins have also shown selective activity towards cancer cells as colicins are more toxic to tumour cells than normal cells (Lancaster *et al*. 2007; Arunmanee *et al*. 2020). In addition, the association of pore forming colicins with colorectal neoplasia was confirmed since it has been found that bacterial strains isolated from large intestinal mucosa in patients with colorectal neoplasia produced higher levels of colicins when compared to similar bacteria found in healthy individuals (Kohoutova *et al*. 2020, Kohoutova *et al*. 2014).

Our previous study reported that colicin N (ColN), a pore-forming colicin, was shown to have selective cytotoxicity towards lung cancer cells (Arunmanee *et al*. 2020, Arunmanee *et al*. 2021). This prompted us to further investigate the cytotoxic effect of ColN on other types of cancer cells including colon, breast, and lung cancer cells. However, it is still unclear whether their mechanisms of action for causing cell death in cancer cells are similar to those in Gram-negative bacteria. ColN consists of three domains namely translocation, receptor binding, and poreforming domains (Vetter *et al*. 1998). All three domains must be combined to cross the bacterial outer membrane and then form an ion channel in the bacterial inner membrane (Cascales *et al*. 2007). In the case of cancer cells without the outer membrane, P domain (also known as toxic domain) alone could be sufficient for causing cancer cell death. Therefore, the cytotoxicity of full-length and truncated ColN (where one or two domains of ColN were deleted) towards cancer cells were also studied. The knowledge gained from this study will support the development and design of colicins as alternatives to conventional cancer therapeutic agents.

## MATERIALS AND METHODS

### Materials

For protein expression and purification, pET3a plasmids encoding c-terminal histidine-tagged ColN was provided by Professor Jeremy H. Lakey, Newcastle University, UK. Luria-Bertani broth and agar were bought from Hardy Diagnostics (Santa Maria, CA, USA). L-(+)-arabinose was obtained from TCI (Tokyo, Japan). Imidazole, DNase I, and ampicillin sodium salt were purchased from PanReac Applichem (Darmstadt, DE, USA). PierceTM protease inhibitor tablets and bicinchoninic acid (BCA) protein assay kit was purchased from Thermo Scientific (Waltham, MA, USA). 10X Phosphate Buffered Saline (PBS), skim milk powder and sodium chloride (NaCl) were purchased from Vivantis Technologies (Selangor, MY) and Ajax Finechem (Seven Hills, NSW 2147, Australia).

For cell culture, DMEM/Ham’s F-12, DMEM high glucose and 1X HEPES were bought from HyClone (Logan, Utah), 10% fetal bovine serum (FBS) from Merck (Darmstadt, Germany), 1% penicilin/streptomycin from Gibco (Gaithersburg, MA, USA). The reagents 3-(4,5-Dimethylthiazol-2-yl)-2,5-diphenyltetrazolium bromide (MTT) were obtained from Abcam (Cambridge, CB2 0AX, UK). Cisplatin and Doxorubicin were bought from Sigma Chemical, Inc. (St. Louis, MO, USA).

### ColN Expression and Purification

C-terminal 6xHis-tagged ColN including ColN-WT, ColN-T, ColN-R, ColN-P, ColN-TR and ColN-RP were constructed in pET3a plasmid. Plasmids encoding ColN gene were transformed into BL21-AI™ One Shot^®^ chemically competent *E. coli* (Invitrogen, USA). The transformed cells from overnight cultures were grown at 37°C in LB broth containing 100 μg mL^−1^ ampicillin. The expression of recombinant proteins was induced by 0.2% (w/v) L-Arabinose at an exponential growth phase (OD_600_ = 0.6 – 0.8). Cells were grown for a further 3 h. The cell pellets were harvested by centrifugation at 8,000xg for 10 min at 4°C. The cell pellets were resuspended in 50 mM sodium phosphate buffer, pH 8.0, 300 mM NaCl, 10 mM imidazole supplemented with containing DNase I and RNase. The resulting mixture was then lysed by sonication and centrifuged at 17,000xg for 20 min at 4°C. The collected supernatant were loaded onto nickel-sepharose HisTrap™ FF affinity column (GE Healthcare Technologist, West Milwaukee, WI, USA) connected to Fast Protein Liquid Chromatography (FPLC) ÄKTA start (GE Healthcare Technologist, West Milwaukee, WI, USA). Bound proteins were eluted by 50 mM sodium phosphate buffer, pH 8.0 with 300 mM NaCl and 250 mM imidazole. Protein-containing fractions were pooled and then dialysed into Phosphate Buffer Saline (PBS) at 4°C overnight. The amount and purity of proteins were assessed by BCA assay and sodium dodecyl sulfate polyacrylamide gel electrophoresis (SDS-PAGE), respectively.

### Western Blot

The proteins separated by SDS-PAGE were transferred onto the nitrocellulose membrane (Merck KGaA, Darmstadt, Germany) using a semi-dry transfer device (Trans-Blot^®^ SD System and PowerPac™ HC Power Supply System, Biorad, USA) at 100 V for 30 min. The membrane was blocked by 5% (w/v) skim milk in PBS for 2 h and then incubated with 1:4000 mouse anti-6xHis antibody (Thermo Fisher Scientific, IL 61105, USA) in 5% (w/v) skim milk in PBS overnight at 4°C. The nitrocellulose membrane was washed three times by PBS with Tween-20 (PBST buffer) for 15 min each. To visualise the bands, the membrane was incubated with 1:4000 goat anti-mouse IgG secondary antibody conjugated with alkaline phosphatase (Seracare KPL, MD, USA) in 5% skim milk for 1.5 h at room temperature. The membrane was washed three times by PBST for 15 min each. The alkaline phosphatase conjugate substrate kit (Biorad, USA) prepared according to the manufacturer procedure was added to the membrane for 2 min to visualise protein bands.

### Secondary Structure Analysis by Circular Dichroism (CD)

The spectra measurement in the range of far-UV at 190–260 nm were carried out at 25°C on a CD spectrophotometer (j-815 CD Spectrophotometer, Jasco, Tokyo, Japan). Proteins at the concentration of 0.4–2.0 mg mL^−1^ in PBS buffer were used. Measurement was used 0.10 cm path length cuvette. The results were converted to molecular ellipticity unit (deg· cm^2^ ·dmol^−1^).

### Cell Culture and Treatment

Cancer cells were purchased from American Type Culture Collection (ATCC, Manassas, VA, USA). The 5 different cancer cells including HCT-116 (Colon cancer cells/ATCC^®^ CCL-247™, USA), HT-29 (Colon cancer cells/ATCC^®^ HTB-38™, USA), MCF-7 (Breast cancer cells/ATCC^®^ HTB-22™, USA), MDA-MB-231 (Breast cancer cells/ATCC^®^ HTB-26™, USA), and A549 (Lung cancer cells/ATCC^®^ CCL-185™, USA). The cells were grown in different medium and supplement, HCT-116, MCF-7 and MDA-MB-231 were grown in DMEM high glucose with 10% FBS, 1% penicillin/streptomycin and 1X HEPES. HT-29 was grown in DMEM/Ham’s F-12 with 10% FBS, 1% penicillin/streptomycin and 1X HEPES. A549 was grown in Ham’s F-12K with 10% FBS, 1% penicillin/streptomycin. Cell culture were incubated at 37°C, 5% CO_2_.

### Cell Viability Assay

Cell viability with and without ColN treatment was monitored by MTT assay and 50 μM cisplatin or 50 μM doxorubicin was utilised as a positive control. Cells were seeded into 96-well plate at density 10,000 cells/well and were grown at 37°C for 24 h. Purified ColN were concentrated by Amicon^®^ Ultra Centrifugal Filters with 10 kDa MWCO and ColN concentrations were determined by BCA assay. Concentrated ColN was then added to the cell culture medium for 72 h at 37°C to at the desired final concentrations. The MTT solution (0.5 mg mL^−1^) was added to each well and incubated for 3 h at 37°C. After removing the culture medium with MTT solution, the formazan was dissolved in 200 μL of DMSO. The absorbance of formazan at 490 nm was measured by a microplate reader (ENVISION, USA). Cell viability is expressed as a percentage of the control group without ColN treatment.

### Statistical Analysis

SPSS Statistics version 25 software was utilised for data analysis and data reported as the mean ± standard deviation. The comparison between groups were carried out by the one-way ANOVA followed by Dunnett’s T3. Statistically significant was set at *p* < 0.05.

## RESULTS

### Recombinant Truncated and Full Length ColN Successfully Expressed in *E. coli*

**Figure 1:**
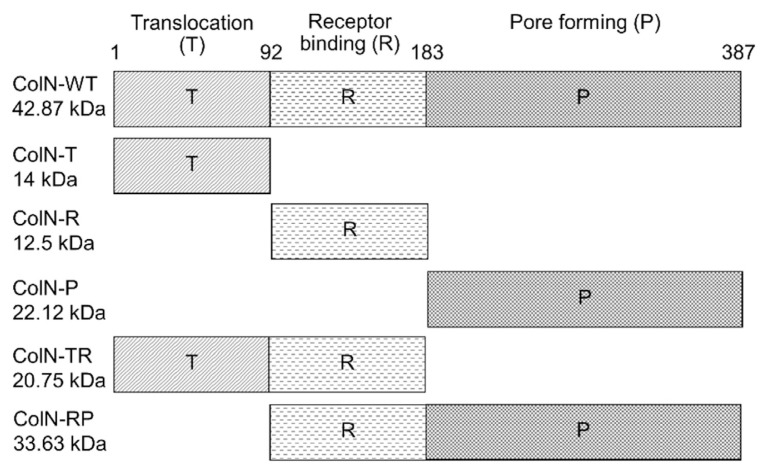
Domain structure and molecular weight of truncated and wild type colicin N in this study. ColN contains translocation (T), receptor binding (R), and pore forming (P) domains.

**Figure 2:**
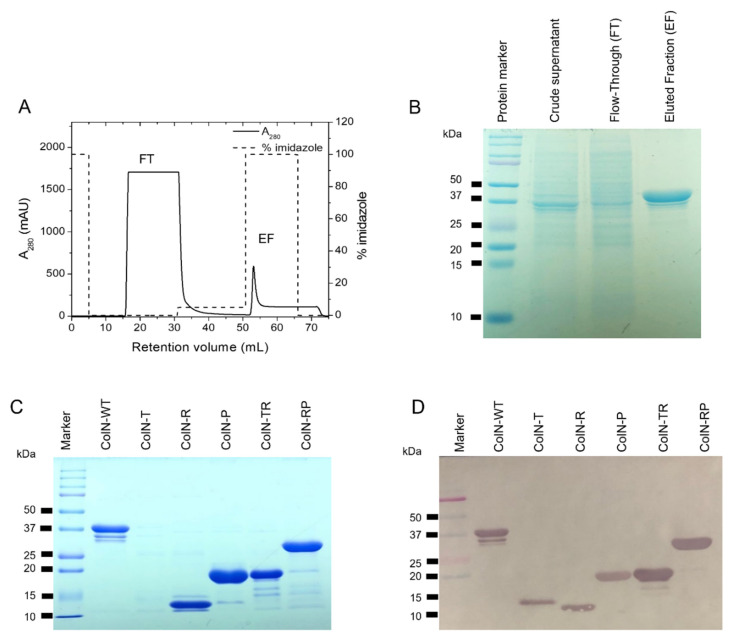
Purification of recombinant ColN with c-terminal histidine tag by affinity chromatography and identification of recombinant ColN (truncated and full length ColN) from *E. coli* expression. (A) Elution profile of ColN-WT using the 1-mL nickel-sepharose HisTrap™ FF affinity column equilibrated with 50 mM sodium phosphate buffer, pH 8.0 with 300 mM NaCl and 10 mM imidazole. An increase from 0% to 100% of the same buffer containing 250 mM imidazole were applied to the column for ColN elution. FT and EF are flow through and elution fractions, respectively. (B) SDS-PAGE analysis of protein-containing fractions obtained from affinity chromatography. (C) SDS-PAGE analysis of purified recombinant ColN. (D) Western blot analysis of recombinant ColN using anti-histag antibodies.

To examine the cytotoxicity of ColN towards cancer cells, five truncated ColN and full-length or wild type ColN (ColN-WT) were expressed in *E. coli* and purified. Full-length ColN (Fig. 1) consists of translocation (T), receptor binding (R) and pore forming domain (P). The truncated ColN as shown in Fig. 1 were constructed with one or two domains of ColN removed. The pET3a plasmid encoding wild type and truncated ColN with c-terminal 6xHis tag were expressed in *E. coli* BL21 AI. As all proteins in this study contain the histidine tag, proteins were loaded on to a Ni column where they were strongly retained. The protein-containing fractions including flowthrough (FT) and elution fractions (EF) were collected. The identity and purity of recombinant proteins in each fraction were initially assessed by SDS-PAGE. Fig. 2A demonstrated the elution profile of ColN-WT purified by FPLC. As expected, most contaminants were not bound to the column and eluted in the FT peak. His tagged proteins were bound to the column and eluted when increasing the concentration of imidazole in running buffer. The SDS-PAGE analysis in Fig. 2B suggested that the purified wild-type ColN in the eluted fraction migrated at approximately 40 kDa corresponding to its theoretical mass of 42 kDa. Likewise, truncated ColN including ColN-T, ColN-R, ColN-P, ColN-TR, and ColN-RP were expressed and purified using the same procedure as ColN-WT. To confirm the identity of these proteins, the analysis of all purified proteins by SDS-PAGE (Fig. 2C) and western blot using anti-histag antibodies (Fig. 2D) indicated that each protein ran at their corresponding size and were bound by anti-histag antibodies. This confirmed that proteins were successfully expressed and purified from *E. coli*.

### Structural Characterisation of ColN Constructs

**Figure 3:**
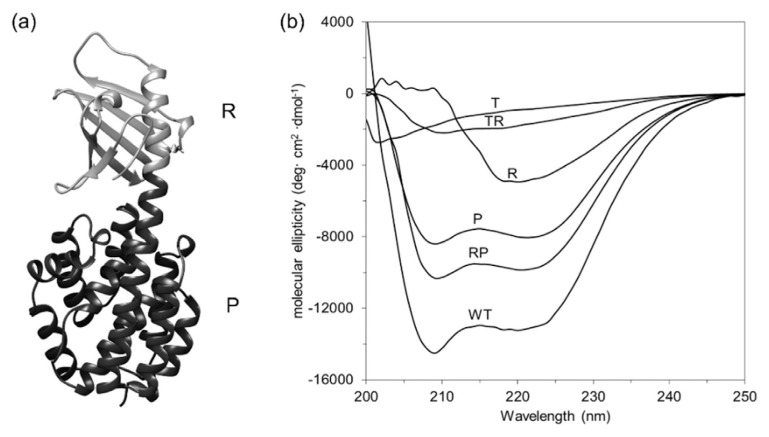
Secondary structure analysis of native ColN and ColN fragments. (A) High resolution structure of native ColN (PDB code: 1A87) showing residues 90–387. Receptor binding (R) and pore forming (P) domains are shown in blue and red, respectively. (B) Far-UV CD spectra of native and truncated ColN. A comparison of their secondary structures was made at 25°C using a cuvette of 0.01 cm path length and protein concentrations of 0.4–2.0 mg mL^−1^. Each spectrum was labelled with the abbreviation of ColN construct.

The high-resolution structure of ColN from residue 90 to 387 (PDB code: 1A87) in Fig. 3A exhibits the beta sheet around a helix in receptor binding domain and 10 helical bundle in pore-forming domain whereas translocating domain classified as intrinsically disordered section was not seen in the high-resolution structure (Vetter *et al*. 1998). Far-UV CD spectroscopy were conducted to measure the secondary structure content of all ColN types. Fig. 3B compares the far-UV CD spectra of truncated and full length ColN. ColN-WT, RP and P gave a typical α spectrum characterised by minima at 222 and 208 nm as the main parts of these proteins are 10 helical bundle. The decrease in molecular ellipticity was observed in ColN-R and TR due to the decreasing proportion of helix. ColN-T showed the weakest signal as it consisted of a poorly defined secondary structure.

### Wild-type ColN Either Inhibits or Promotes the Viability of Cancer Cells

**Figure 4:**
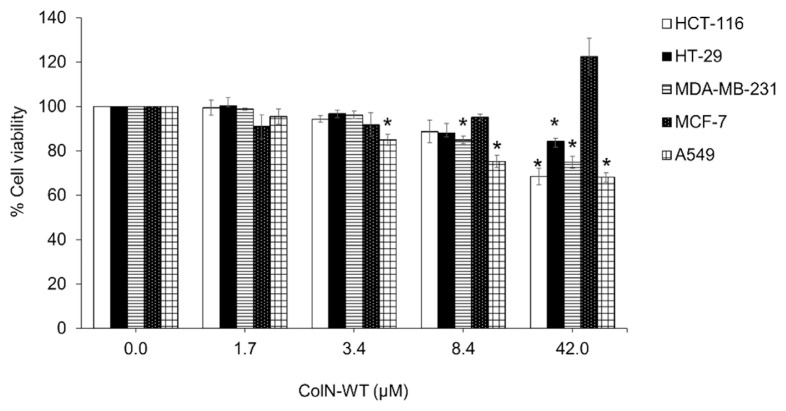
Effect of ColN-WT on viability of breast (MCF-7 and MDA-MB-231), lung (A549), and colon cancer cells (HT-29 and HCT-116). Cells were treated with vehicle control, different concentrations of ColN-WT, and positive control (50 μM cisplatin or 50 μM doxorubicin) for 72 h. MTT assay were carried out to measure cell viability. Data are reported as the mean ± standard deviation. Statistical significance was set at *p ≤ 0.05.

Five different cancer cell lines were selected for performing the *in vitro* cytotoxicity of ColN-WT. HCT-116, HT-29, MCF-7, MDA-MB-231, and A549 cells were treated with different concentrations of ColN-WT for 72 h. The MTT assay was used to determine the cell viability of untreated and treated cells. The findings in Fig. 4 showed that ColN-WT inhibited growth of cancer cells including HCT-116, HT-29, MDA-MB-231, and A549 cells, in a concentration dependent manner. The ColN-WT concentration of 3.4 μM began to cause a slightly decrease in %cell viability in MDA-MB-231 and A549 (*P < 0.05) whereas the ColN-WT concentration of 8.4 μM started to suppress the growth of HCT-116 and HT-29 (*p < 0.05). The growth inhibition was 31.5% in HCT-116, 15.8% in HT-29, 25.1% in MDA-MB-231, and 31.9% in A549 cells after the addition of ColN-WT (1.7 – 42 μM). However, the promotion of cancer cell growth was observed in MCF-7 cells and growth promotion were 22.5% in MCF-7 cells after the treatment with ColN-WT (1.7 – 42 μM). As presented in Fig. 4, the maximal effect was obtained at a ColN-WT concentration of 42 μM in HCT-116 and A549 cells. Based on this experiment, HCT-116 cells were chosen for subsequent tests. The cytotoxicity of ColN in colon cancer cells was worth further exploration as there were evidence supporting the correlation between increase in production of pore-forming colicins in large intestinal mucosa and colon cancer patients (Kohoutova *et al*. 2014; Kohoutova *et al*. 2020).

### Some ColN Constructs Showed Weak Inhibition of Cancer Cell Proliferation

**Figure 5:**
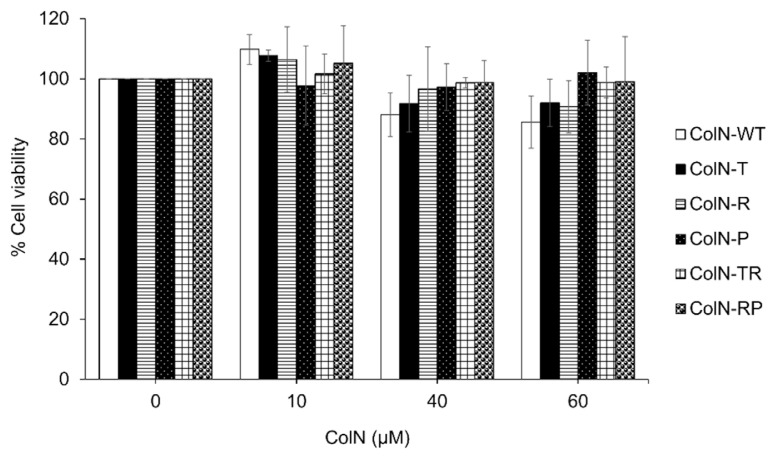
Effect of ColN domains on viability of colon cancer cells (HCT-116). Vehicle control, different concentrations of ColN, and positive control (50 μM cisplatin or 50 μM doxorubicin) were added and incubated with cells for 72 h. MTT assay were carried out to measure cell viability. Data are reported as the mean ± standard deviation. Statistical significance was set at *p ≤ 0.05.

Truntcated (single domains or combination of two domains) and full length ColN were produced in *E. coli* to assess their inhibitory effect on cancer cells. To screen for the cytotoxic effect between ColN-WT and various types of truncated ColN, HCT-116 cells were treated with full-length and truncated colicin N at 0 – 60 μM and incubated for 72 h at 37°C. The results (Fig. 5) showed that no significant cytotoxic effect was observed in HCT-116 cells at the highest concentration of all ColN. ColN-WT, ColN-T and ColN-R seemed to slightly decrease the growth of cancer cells.

## DISCUSSION

In this work, we explored a novel therapeutic protein, colicin N, which is categorised into the family of pore forming toxins. The data presented here provide additional information about the cytotoxicity of ColN against several cancer cells and the essential ColN domains.

### Effect of Full Length ColN on Various Types of Cancer Cells

#### Colon cancer cells (HT-29 and HCT-116)

ColN-WT, a pore forming toxins from *E. coli*, was tested for cytotoxicity. Apart from the bactericidal activity in *E. coli*, the specific cytotoxicity of ColN-WT against lung cancer cells was recently shown (Arunmanee *et al*. 2020). Other common cancer cell lines including colon, breast and lung cancer cells were chosen to test the cytotoxicity of ColN in this study. The proliferation of colon cancer cells (HT-29, and HCT-116) decreased in the presence of ColN-WT and HCT-116 was more sensitive to this protein than HT-29. This finding is in good agreement with previous studies indicating that these two cell lines showed different responses to chemotherapeutic treatment in normal and hypoxia conditions (Yao *et al*. 2005; Jang *et al*. 2019). HT-29 and HCT-116 bear different mutations and are classified as p53-deficient and p53-positive cell lines, respectively (Rodrigues *et al*. 1990; Bhattacharyya *et al*. 1994). Effects of other pore-forming colicins, such as colicin A and E1, on HT-29 were also reported indicating that HT-29 was insensitive to colicin E1 but sensitive to ColA (Chumchalova & Smarda 2003).

#### Breast cancer cells (MCF-7 and MDA-MB-231)

Meanwhile, this study suggests that ColN-WT at high concentration slightly inhibited proliferation of breast cancer cell line (MDA-MB-231) but significantly promoted proliferation of breast cancer cell line (MCF-7). The main characteristics of MCF-7 cells is the over-expression of epidermal growth factor receptor (Levenson & Jordan 1997) whereas that of MDA-MB-231 cells is triple negative cells (Gest *et al*. 2013). In contrast to ColN-WT, ColA and E1 inhibited growth of these breast cancer cells *in vitro* (Chumchalova & Smarda 2003). Furthermore, our initial results showed that the presence of ColN-WT at high concentration elevated cell viability in rat pancreatic acinar cells (AR42J) and gastric carcinoma cell (NCI-N87) (Pitchayakorn *et al*. 2020).

#### Lung cancer cells (A549)

In the case of lung cancer cells (A549), the exposure of ColN-WT to A549 cells caused the decrease in cell viability in a concentration dependent manner. The same effects were observed in other lung cancer cell lines. We previously demonstrated that H23, H292 and H460 lung cancer cells were also sensitive to ColN-WT (Arunmanee *et al*. 2020).

### Effect of Truncated ColN Domains on Colon Cancer Cells

To compare the mechanism of action of ColN towards *E. coli* with cancer cells, we resorted to testing ColN in the absence of one or two domains. All three domains of ColN are essential for efficiently mediating cell killing in *E. coli*. The R domain interacts with the receptor on the surface of *E. coli* cells. This is followed by the translocation of ColN from *E. coli* outer membrane towards the inner membrane which is triggered by the T domain. In the final step, P domains or toxic domains are inserted into the bacterial membrane to form ion channels causing cell leakage (Vetter *et al*. 1998). As there is only one membrane in cancer cells, we speculated that the mechanism of action of ColN against cancer cells would need only the P domain. Therefore, a series of truncated ColN with a deletion of one or two domains of ColN were treated with HCT-116 to observe their differences in cytotoxic effects. HCT-116 was not significantly sensitive to all ColN but ColN-WT slightly decrease the growth of HCT-116 when compared to other types of ColN. This implied that the cytotoxicity of ColN required the function of all domains. The binding of P domain to cell membrane is accomplished by the unfolding of ColN (Ridley *et al*. 2010). Dover *et al*. (2000) showed that pre-treated ColN with detergents enhanced membrane insertion. In contrast to ColN, ColA was unfolded at low pH leading to membrane insertion (Evans *et al*. 1996). We propose that the acidic environment of cancer cells promoted the membrane insertion of ColA but not ColN. This could be the reason why cancer cells were more sensitive to ColA compared to ColN. The absence of one of the domains could also affect this unfolding event and ultimately disrupt the function of ColN towards the cancer cells.

## CONCLUSION

In summary, the mild toxicity of ColN varies depending on the type of cancer cells whereas the viability of certain cancer cells was elevated due to the treatment of high concentration of ColN. The promotion of cell viability caused by ColN treatment still requires further investigation. However, P domain or toxic domain of ColN alone is not effective enough to inhibit the proliferation of cancer cells. This may indicate that the function of ColN to inhibit the growth of cancer cells is only achieved by the association of all domains. The future direction for this research would be to investigate the cytotoxic effect of ColN against tumors in animal models in order to support the *in vitro* cytotoxic studies of this protein. Moreover, the combination of other chemical drugs with lower doses of ColN should be investigated to observe whether reduced concentrations of ColN would still be effective in inhibiting cancer cell proliferation.
